# Autologous Platelet-Rich Fibrin in the Treatment of Refractory Macular Holes

**DOI:** 10.1155/2019/6054215

**Published:** 2019-11-06

**Authors:** Arif Koytak, Fadime Nuhoglu, Havvanur Bayraktar, Rukiye Ercan, Hakan Ozdemir

**Affiliations:** Bezmialem Vakif University, Faculty of Medicine, Department of Ophthalmology, Istanbul, Turkey

## Abstract

**Purpose:**

To describe a novel method for the treatment of refractory macular holes.

**Methods:**

Two case reports on the use of autologous platelet rich fibrin (PRF), followed by sulfur hexafluoride gas tamponade to facilitate closure of refractory macular holes.

**Results:**

Macular holes were succesfully closed within a week in both cases. Best corrected Snellen visual acuities improved from counting fingers to 0.16 in the first case, and from 0.05 to 0.2 in the second case. No complication occurred during or after the procedures.

**Conclusion:**

The use of autologous PRF seems to be a safe and effective alternative method for the treatment of refractory macular holes. Further experience and studies are required to assess the value of autologous PRF in the management of challenging macular hole cases of different etiologies. To the best of our knowledge, this is the first use of autologous PRF in the treatment of macular holes.

## 1. Introduction

Full thickness macular holes (FTMH) are not infrequent and more than 90% of them are successfully treated with a single surgery [1]. Current surgical technique of choice consists of pars plana vitrectomy and internal limiting membrane (ILM) peeling, followed by air-gas exchange and postoperative face-down positioning. However, 5–10% of FTMHs which fail to close with a single surgery require further interventions [2].

Repeat air-gas exchange, sometimes with a longer acting gas, is the first option in the management of refractory macular holes. However, only 46.7% of these FTMHs are found to be closed following reoperation [3]. Success rates in chronic and large holes are even lower.

Several surgical techniques are developed and tried for the management of refractory macular holes. These include relaxing retinotomy, free and inverted ILM flaps, posterior lens capsular flap, autologous neurosensorial retinal flap, and foveal hydrodissection. Autologous blood products such as simple blood clot and platelet rich plasma, [4–6] as well as several tissue glues comprising nonautologous blood products and synthetic molecules are also tried for facilitating hole closure [7–12].

Platelet rich fibrin (PRF) is a leukocyte and platelet rich concentrate obtained from venous blood through a simple and inexpensive technique developed by Choukroun et al. which provides a strong and elastic fibrin matrix. In contrast to other autologous blood products, this concentrate is produced without any anticoagulants or gelifying agents [13, 14].

Here, we describe the closure of two refractory macular holes with the use of PRF. To the best of our knowledge, this is the first use of PRF in the treatment of FTMH.

## 2. Case 1

A 68-year-old woman had a history of pars plana vitrectomy with ILM peeling and silicone oil tamponade for the treatment of concommitant rhegmatogenous retinal detachment and FTMH in her left eye before 6 years. She had her retinal detachment repaired, but the macular hole persisted despite long term tamponade with silicone oil. A year after the initial one, she had a second surgery using short term gas tamponade with sulfur hexafluoride gas (SF_6_), but the hole failed to close. A third attempt to close the macular hole was carried out a year after, with carbon hexafluoride gas (C_3_F_8_). Despite her strict obedience to face down positioning, the hole failed to close.

After three operations that failed, the patient did not prefer to have further interventions and remained on regular follow-up visits for about 4 years. During this time period no significant anatomical and functional change occurred in her left eye.

In her last visit, she had a best corrected visual acuity (BCVA) of counting fingers (CF) in her left vitrectomized eye which had a chronic large macular hole with a base daimeter of 1697 micrometers. Upon proposal, she has accepted a fourth surgery with autologous PRF application.

The surgical technique included four ports pars plana approach for the application of a round button of PRF to the macular hole, followed by fluid-air and air-SF_6_ gas exchange. The patient was ordered to have face down positioning for three days following the operation.

The PRF button was cut with scissors from a PRF membrane which was prepared with Choukrun's procedure described elsewhere [7, 8]. In short, this procedure consisted of centrifuge of autologous blood drawn immediately before the operation, followed by removal of the fibrin clot from the test tube and its compaction with a metal press board. The PRF button was slightly larger than the macular hole, permitting complete plugging of the hole (see video, Supplementary [Supplementary-material supplementary-material-1], which summarizes surgical steps).

**Figure 1:**
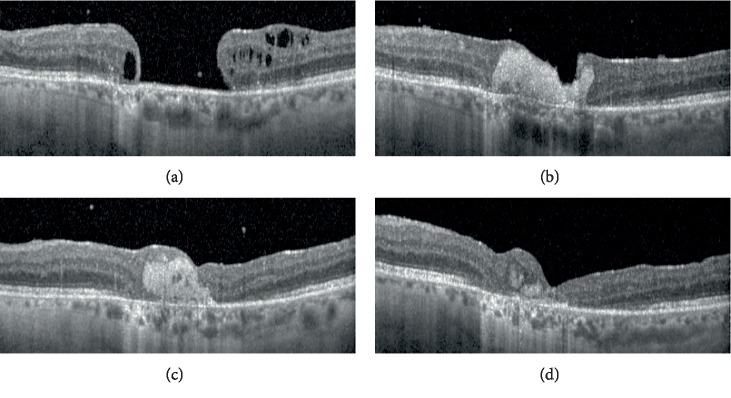
Optical coherence tomographic images of the Case 1 before the operation (a), and one week (b), one month (c), and 3 months (d) after the operation.

The week after the operation, the hole was shown to be completely sealed by the PRF button on optical coherence tomographic (OCT) images. The button was resorbed slowly until the third postoperative month, and replaced by neuroglial proliferation which closed the hole completely (Figure 1).

Despite the chronic course of the pathology and low initial visual acuity, the Snellen BCVA improved to 0.16 in the last control visit. No complication occurred during or after the procedure.

## 3. Case 2

A 79 years old woman with a FTMH in her left eye was treated with pars plana vitrectomy with ILM peeling and SF_6_ gas tamponade. She had a Snellen BCVA of 0.05 in her left eye and the hole diameter was 1581 micrometers prior to the surgery. As the first operation failed to close the FTMH, she had a second operation consisting of four ports pars plana approach for the application of a round button of PRF to the macular hole, followed by fluid-air and air-SF_6_ gas exchange. Three days strict face down positioning was ordered following the operation.

**Figure 2:**
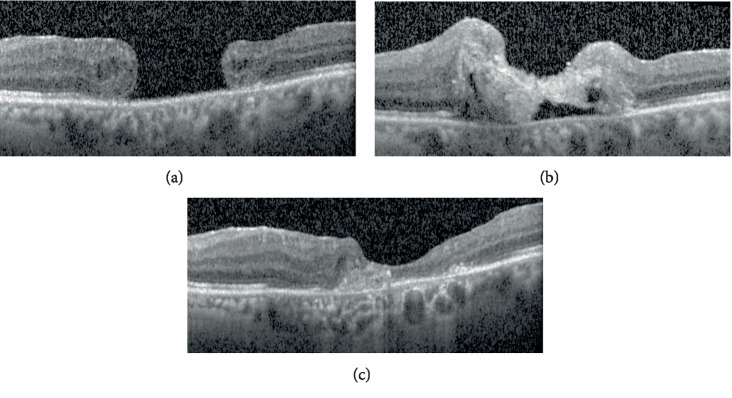
Optical coherence tomographic images of the Case 2 before the operation (a), and one week (b), and 3 months (c) after the operation.

The week after the application of PRF, the hole was shown to be closed on OCT (Figure 2). The PRF button was slowly resorbed and the hole remained closed until the last visit at the fourth postoperative month. The Snellen BCVA improved from 0.05 to 0.2. The surgery and postoperative period was free of complications.

## 4. Conclusion

Pars plana vitrectomy with ILM peeling, followed by gas tamponade is the initial treatment of choice for the majority of uncomplicated macular holes and has been proven to be successuful in more than 90% of cases. Large, highly myopic, [15] traumatic or chronic macular holes are less likely to close with a single procedure and several alternative methods are proposed for such refractory cases. The use of inverted or free ILM, posterior lens capsule, or autologous neurosensorial retinal flaps, relaxing retinotomy, foveal hydrodissection, and autologous serum are among alternatives suggested to be effective. Also, several blood products (autologous, allogeneic, or xenogeneic) and synthetic materials are studied as injectable tissue glues to seal retinal tears or macular holes in both animal experiments and human eyes [7–12]. Although most of these tissue glues are proven to be effective sealants, they gained limited popularity because of the complexity of their preparation and handling steps and their limited availability as expensive commercial products. Also, these glues which comprise nonautologous components such as allogeneic fibrinogen and thrombin, bovine thrombin, or synthetic molecules such as cyanoacrylate are suggested to carry risks of inflammation and/or retinal toxicity resulting in damage to adjacent healthy retina, epiretinal proliferation, retinal contraction, and subnormal visual recovery [7, 16–19].

Unlike all other injectable blood products that are studied for similar purposes, autologous PRF is a solid and elastic membrane which is easy to procure and manipulate at almost no cost. It acts as a resorbable plug, rather than a tissue glue. It is readily absorbed and totally replaced by the neuroglial tissue. And because it does not contain any nonautologous molecule, it seems not to provoque any unwanted inflammatory or toxic reactions. Autologous PRF, besides providing a dry medium and a solid framework for healing, acts as a slow-release fibrin matrix which contains several growth factors and cytokines as well as mesenchymal stem cells which are believed to promote healing process [20].

Our experience with two refractory large macular hole cases which achieved complete closure revealed a new alternative for the management of macular holes. During the follow-up period, we did not encounter any complication or adverse reaction related to the procedure. Anatomical restoration and functional improvement was achieved in both cases. To our knowledge, this is the first report about the use of autologous PRF in the treatment of FTMH.

The use of autologous PRF could be seen as a safe and effective alternative method for the treatment of challenging large, highly myopic, chronic, traumatic, recurrent, or pediatric macular hole cases. PRF buttons could also be helpful as temporary plugs in the surgical management of optic pits. Further experience and studies are required to assess the value of PRF in the management of each specific entity mentioned above.

## Additional Points

We report two successful cases on the use of autologous platelet rich fibrin (PRF) to facilitate closure of refractory macular holes. The use of autologous PRF seems to be a safe and effective alternative method for the treatment of refractory macular holes.

## Supplementary Materials

Supplementary Materials There is only one supplemental file which is a .wmv video file summarizing the surgical steps.
